# Understanding Strategy of Nitrate and Urea Assimilation in a Chinese Strain of *Aureococcus anophagefferens* through RNA-Seq Analysis

**DOI:** 10.1371/journal.pone.0111069

**Published:** 2014-10-22

**Authors:** Hong-Po Dong, Kai-Xuan Huang, Hua-Long Wang, Song-Hui Lu, Jing-Yi Cen, Yue-Lei Dong

**Affiliations:** Research Center for Harmful Algae and Marine Biology, Key Laboratory of Eutrophication and Red Tide Prevention of Guangdong Higher Education Institutes, Jinan University, Guangzhou, China; University of Connecticut, United States of America

## Abstract

*Aureococcus anophagefferens* is a harmful alga that dominates plankton communities during brown tides in North America, Africa, and Asia. Here, RNA-seq technology was used to profile the transcriptome of a Chinese strain of *A. anophagefferens* that was grown on urea, nitrate, and a mixture of urea and nitrate, and that was under N-replete, limited and recovery conditions to understand the molecular mechanisms that underlie nitrate and urea utilization. The number of differentially expressed genes between urea-grown and mixture N-grown cells were much less than those between urea-grown and nitrate-grown cells. Compared with nitrate-grown cells, mixture N-grown cells contained much lower levels of transcripts encoding proteins that are involved in nitrate transport and assimilation. Together with profiles of nutrient changes in media, these results suggest that *A. anophagefferens* primarily feeds on urea instead of nitrate when urea and nitrate co-exist. Furthermore, we noted that transcripts upregulated by nitrate and N-limitation included those encoding proteins involved in amino acid and nucleotide transport, degradation of amides and cyanates, and nitrate assimilation pathway. The data suggest that *A. anophagefferens* possesses an ability to utilize a variety of dissolved organic nitrogen. Moreover, transcripts for synthesis of proteins, glutamate-derived amino acids, spermines and sterols were upregulated by urea. Transcripts encoding key enzymes that are involved in the ornithine-urea and TCA cycles were differentially regulated by urea and nitrogen concentration, which suggests that the OUC may be linked to the TCA cycle and involved in reallocation of intracellular carbon and nitrogen. These genes regulated by urea may be crucial for the rapid proliferation of *A. anophagefferens* when urea is provided as the N source.

## Introduction

Brown tides are caused by the pelagophyte *Aureococcus anophagefferens*, which is a small (∼2–3 µm) eukaryotic phytoplankton. This harmful algal bloom (HAB) has plagued many coastal ecosystems in the Eastern United States and South Africa since its discovery in 1985. Although brown tides do not produce harmful toxins, these tides still decimate fisheries and seagrass beds because of toxicity to bivalves and extreme light attenuation, respectively [1]. Recently, large-scale brown tides have been reported in China, which occurred in early summer for three consecutive years from 2009 to 2011 in the coastal waters of Qinhuangdao, China [2]. This report shows that brown tides are expanding and spreading to other oceanic regions because of anthropogenic activities. It is important to determine the causes of brown tides.

*A. anophagefferens* often bloom in periods when levels of dissolved inorganic nitrogen (DIN) are low and dissolved organic nitrogen (DON) concentrations are elevated [3]. *A. anophagefferens* is able to utilize a variety of DON compounds, which may facilitate its growth as both carbon and nitrogen sources [4], [5], [6]. In addition, it has been shown that *A. anophagefferens* has a significantly greater uptake capacity for urea than for other N sources that were tested, including nitrate, glutamic acid, and ammonium [4], and its growth increases as the DON:DIN ratio increases. These results suggest that blooms of *A. anophagefferens* may be related to the preferred utilization and high uptake of DON by *A. anophagefferens*. However, Pustizzi et al. (2004) found that although low light cultures of *A. anophagefferens* with urea have higher growth rates than those cultures without urea, the growth on urea is not significantly faster than the growth on nitrate [7]. It is assumed that uptake rates of urea by *A. anophagefferens* may be separate from the actual assimilation of urea. These results indicate that the utilization of urea by *A. anophagefferens* is complicated and probably affected by other environmental factors, such as nutrient levels and light intensity. Based on the above studies, it is proposed that the rapid growth of *A. anophagefferens* on urea may be associated with the fixation of urea-C. In-depth studies are required to reveal the real mechanism underlying urea utilization by *A. anophagefferens.*

Organic N sources in the ocean are diverse and presumably include urea, amines, peptides, proteins, nucleic acids, amino sugars, and amides. *A. anophagefferens* has order of priority in options of different N sources. These options are supported by the observation that the fastest growth is observed in cultures that are grown on urea, followed by acetamide, nitrate, ammonium, and formamide [8]. The characterization of a cDNA library and gene expression suggests that *A. anophagefferens* can assimilate eight different forms of N, and growth on different N sources elicits an increase in the relative expression of corresponding N transporters [8]. Recently, genome analysis found that, relative to competing phytoplankton, *A. anophagefferens* is enriched in genes encoding enzymes that degrade organic nitrogen compounds and transporters that are specific for a diverse set of organic nitrogen compounds [9], [10]. These studies suggest that *A. anophagefferens* has a greater capacity to use organic nitrogenous compounds compared with its competitors. More recently, transcriptome analysis found that *A. anophagefferens* cells express and regulate a suite of genes that are related to organic nitrogen acquisition/metabolism under nitrogen depletion, which further supported the conclusion that *A. anophagefferens* can metabolize reduced organic forms of N [11].

The recently developed RNA-seq technology [12] has made genome-wide transcript analyses both sensitive and quantitative. Additionally, it has been demonstrated that RNA-seq is an excellent genome-scale platform for analyzing transcript levels [13]. In this study, we report the use of RNA-seq technology to examine transcriptomic differences in a Chinese strain of *A. anophagefferens* that was grown on urea, nitrate, or a mixture of urea and nitrate, and that was under N-replete, limited and recovery conditions. These RNA-seq studies have produced large, quantitative data sets for transcript abundance in *A. anophagefferens* by mapping RNA-seq reads to its gene models. The data strongly suggest significant differences in key cellular metabolic pathways, such as N transport and metabolism, the ornithine-urea cycle (OUC), and the tricarboxylic acid (TCA) cycle, among the different experimental groups. To our knowledge, this study is the first to find OUC activity in *A. anophagefferens.*

## Materials and Methods

### Algal strain

*A. anophagefferens* was collected from the coastal water of Qinhuangdao in the Bohai Sea, China on June 20, 2012 at station X01 (119°37.911′ E, 39°54.111′ N). The station X01 was located in a region that was experiencing a brown tide on that date. The oceanic region is open to the public and no specific permissions are required for sampling. *A. anophagefferens* cells were isolated using capillary pipettes under an inverted microscope and subsequently cultures from a single cell were established. The culture strains were maintained in sterilized natural seawater. Here, it should be pointed out that the field sampling did not involve endangered or protected species.

### Culture conditions

The cultures were grown in 2-L flasks with 1 L of artificial seawater medium [14], which was enriched with f/2 nutrients, with nitrate as the N source (882 µmol L^−1^ NO_3_^−^ and 36.3 µmol L^−1^ PO_4_^3−^). Vitamins (thiamine, biotin and B_12_) were sterile filtered and added to the media after autoclaving. The cultures were grown at 18°C on a 12:12 h light: dark cycle under cool-white fluorescence lights (100 µmol photon m^−2^ s^−1^), and the rate of growth was measured by monitoring in vivo fluorescence using a Turner Designs Model 10 Fluorometer (Turner Designs, CA, USA). These cultures were harvested during the late exponential growth phase and then inoculated into three different artificial seawater media with three different sources of N, including nitrate (882 µmol L^−1^ final concentration), urea (441 µmol L^−1^ final concentration), and nitrate + urea (Mixture N, 441 µmol L^−1^ nitrate, 220 µmol L^−1^ urea, final concentration). All cultures were grown in triplicate. The cells were harvested at the onset of the stationary phase by centrifugation (8000 × g for 10 min), covered with RNAlater solution (Sigma) and stored at −80°C until the RNA was extracted.

For nitrogen-limited and recovery experiments, cells from an exponential culture grown in f/2 media with urea as the N source (882 µmol N. L^−1^ final concentration) were collected by centrifugation (6000 × g for 5 min), washed once with nitrogen-free media and then inoculated in N-replete (441 µmol L^−1^ urea) and N-limited (20 µmol L^−1^ urea) media, respectively. The growth of the cultures was monitored by measuring in vivo fluorescence. The cells were harvested by centrifugation (8000 × g for 10 min) after 6 days in the stationary phase when nitrogen was depleted in the N-limited media. The remaining nitrogen-starved cultures were divided into two parts and then received additions of either 882 µmol L^−1^ NO_3_^−^ or 441 µmol L^−1^ urea. RNA samples were collected at 24 h.

### Chlorophyll fluorescence measurements

The parameters *Fv/Fm* and the rapid light curves for ETR (electron transport rate) were determined using a Phyto-PAM Phytoplankton Analyzer (Walz, Germany). The culture was dark-acclimated for 15–20 min before determining *Fv/Fm*. The rapid light curves for ETR were measured under different PAR levels. Light-saturated ETR, ETRmax and the efficiency of the electron transport were analyzed from light curves of ETR [15].

### Analysis of nutrient

Culture media were collected every other day and filtered through a GF/F filter. All filtrate samples were stored at −20°C until analysis. Urea concentration was colorimetrically determined using the diacetyl monoxime method by Rahmatullah and Boyde [16]. Nitrate were measured using flow injection analyzer LACHAT QC 8500 (HACH, USA) following spectrophotometric method [17].

### Total RNA extraction and Illumina sequencing

Total RNA was extracted from frozen cell pellets using Trizol reagent (Invitrogen, CA, USA) according to the manufacturer's instructions. RNA concentrations were determined from A_260_ nm, and its purity was evaluated by the A_260_ to A_280_ nm ratio. The integrity of the total RNA was assessed using an Agilent 2100 Bioanalyzer. RNA-seq libraries were constructed following an Illumina gene expression sample preparation kit. Briefly, total RNA (5–10 µg) was treated with RNase-free DNase I. Poly(A) mRNA was isolated using oligo(dT) magnetic beads and then fragmented into short fragments (approximately 200 bp). The first-strand synthesis of cDNA was performed using random hexamer-primed reverse transcription. The second-strand synthesis was performed by adding the first strand cDNA synthesis reaction to a second strand reaction mix consisting of first strand buffer, second strand buffer, a dNTP mix, RNase H (Invitrogen) and DNA polymerase I (Invitrogen). The double stranded cDNA was subsequently purified using magnetic beads. End reparation and 3′-end single nucleotide A addition was performed. Then, the cDNA fragments were connected with sequencing adaptors and were enriched by PCR amplification. Finally, the library was sequenced in BGI-tech (Shenzhen) using an Illumina HiSeq 2000 sequencer. RNA-seq raw data have been deposited in the NCBI Gene Expression Omnibus (GEO) database with experiment series accession number GSE60576.

### Analysis of differentially expressed genes

The raw image data were converted into sequence data by base calling which are defined as raw reads. These raw reads had a sequencing length of 50 bp. To obtain high-quality reads, the raw reads were filtered to remove reads with adaptor sequence, low-quality reads and reads with high percentage of unknown bases using BGI-tech's in-house software SOAPnuk. All processed clean reads were mapped to the reference genome and transcript of *A. anophagefferens* using the program SOAPaligner/SOAP2 (version 2.21)[18], respectively, which were downloaded from http://genome.jgi.doe.gov/Auran1/Auran1. This alignment allowed no more than two mismatches. In addition, considering that *A. anophagefferens* genome just contains 1185 scaffolds and lacks prediction of gene models, the reads that mapped to the genome were not used for quantification analysis. In contrast, the reference transcript includes a total of 11501 gene models built by homology to known proteins from other model organisms and ab initio gene predictions as well as from available *A. anophagefferens* EST and cDNA data, so the differential gene expression analysis was performed on the reads that mapped to the transcript. The number of clean reads for each gene was calculated and then normalized to RPKM (number of transcripts per million clean reads), which is related to the read number with gene expression levels [19]. Fold changes in the differential gene expression between conditions were calculated using the log_2_ ratio of RPKM.

The significance of differentially expressed genes between two experimental groups (p-value) were performed following a published method ([20]. A false discovery rate (FDR)≦ 0.001 and an absolute value of log_2_ ratio ≧1 were used as cutoffs to judge the significance of gene expression differences [21].

### Annotation

No function annotation in the transcript which was used as reference genes is provided. In order to annotate these mapped genes, we performed a BLAST search against the non-redundant (NR) database in NCBI with an e-value cut-off of 1e^−5^. Those best hits with specific function whose score is the highest and e-value > 1e^−5^ were chosen. The Blast2GO program was used to obtain the Gene ontology (GO) annotation of the genes.

## Results

### Physiological responses to different N sources, N-limitation and recovery

**Figure 1:**
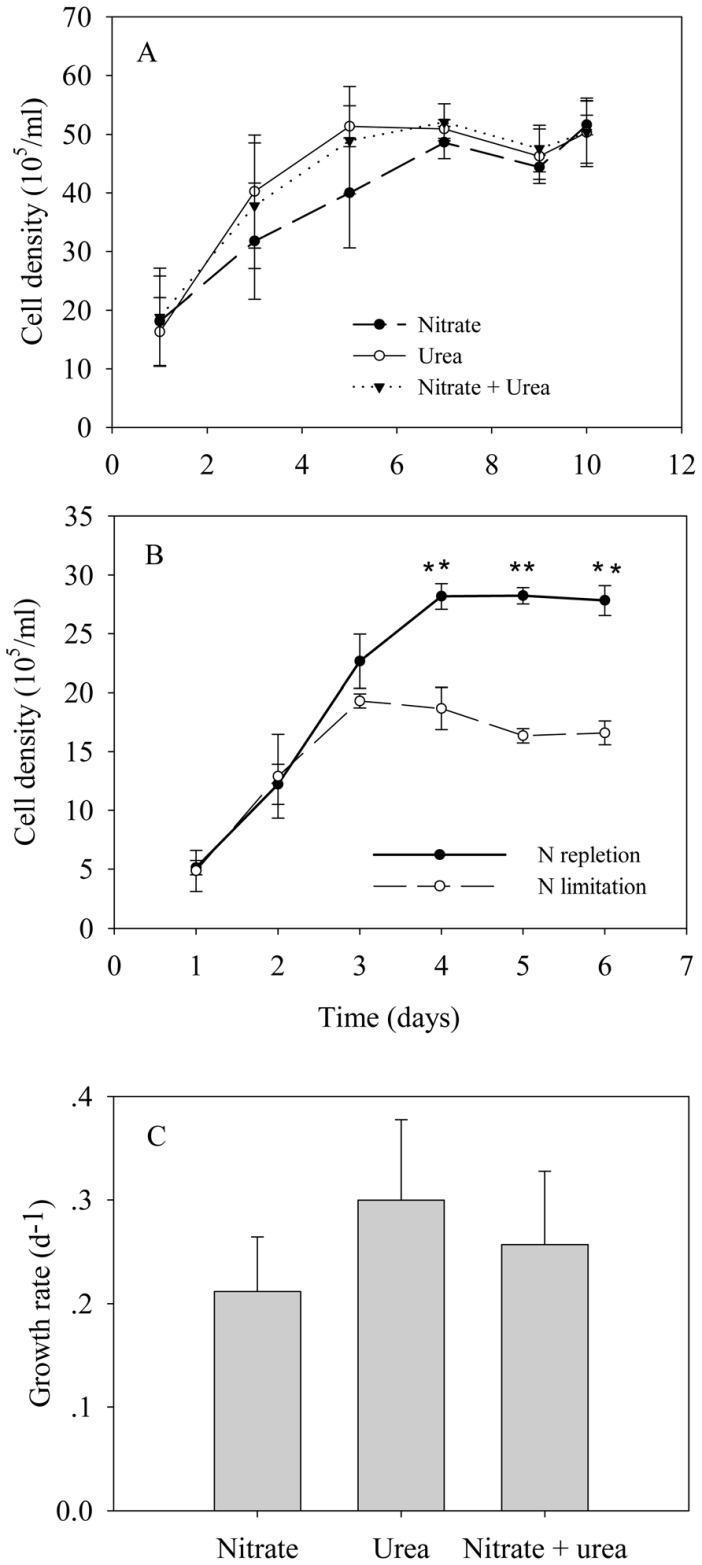
Cell density as a function of culture time. A) Growth of *A. anophagefferens* grown on urea, nitrate, and a mixture of urea and nitrate. B) Growth of *A. anophagefferens* under nitrogen-replete and limited conditions. Cell density under nitrogen-limited condition was compared with that under nitrogen-replete condition. Significance values were expressed as follows: * *P* <0.05, ** P <0.001. C) Growth rates of *A. anophagefferens* grown on urea, nitrate, and a mixture of urea and nitrate. Error bars represent standard deviation of the mean for the three biological replicates.

**Figure 2:**
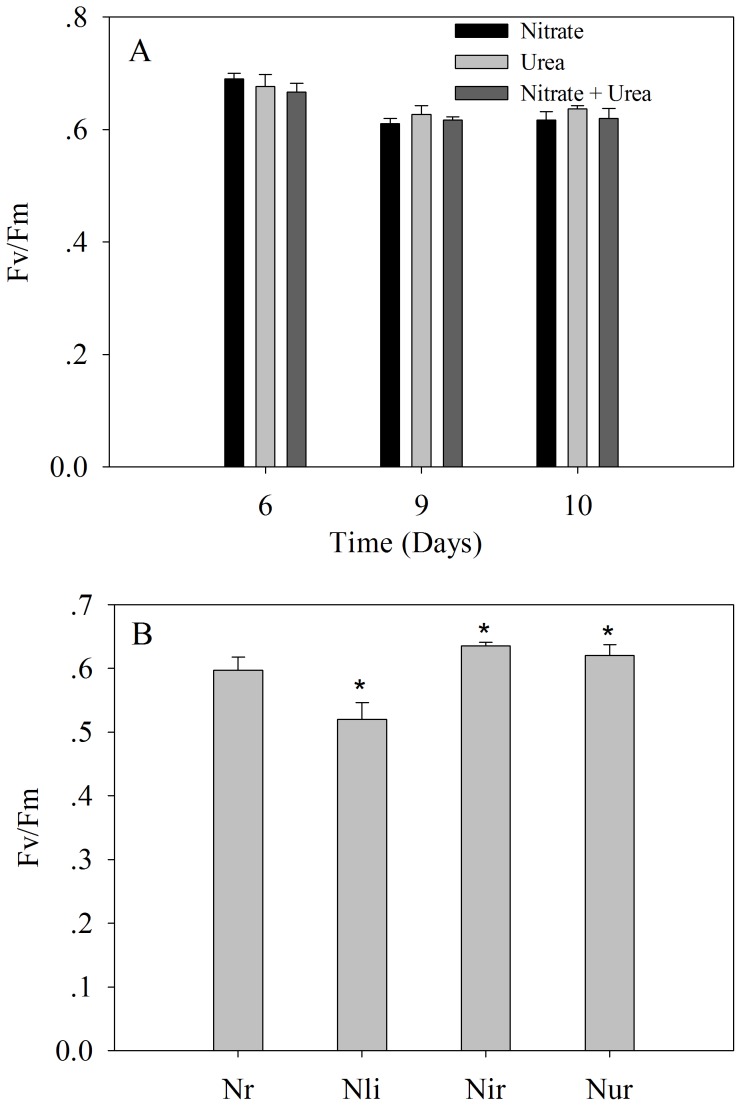
Photosystem II efficiency (Fv/Fm). A) *A. anophagefferens* cultures grown on urea, nitrate, and a mixture of urea and nitrate at the 6-, 9- and 10-day sampling points; B) *A. anophagefferens* cultures grown under nitrogen-replete (Nr), limited (Nli) and recovery conditions. Nir and Nur represent nitrate and urea addition, respectively. Nli was compared with Nr while Nir and Nur were compared with Nli. Significance values were expressed as follows: * *P* <0.05, ** P <0.001. Error bars represent standard deviation of the mean for the three biological replicates.

**Figure 3:**
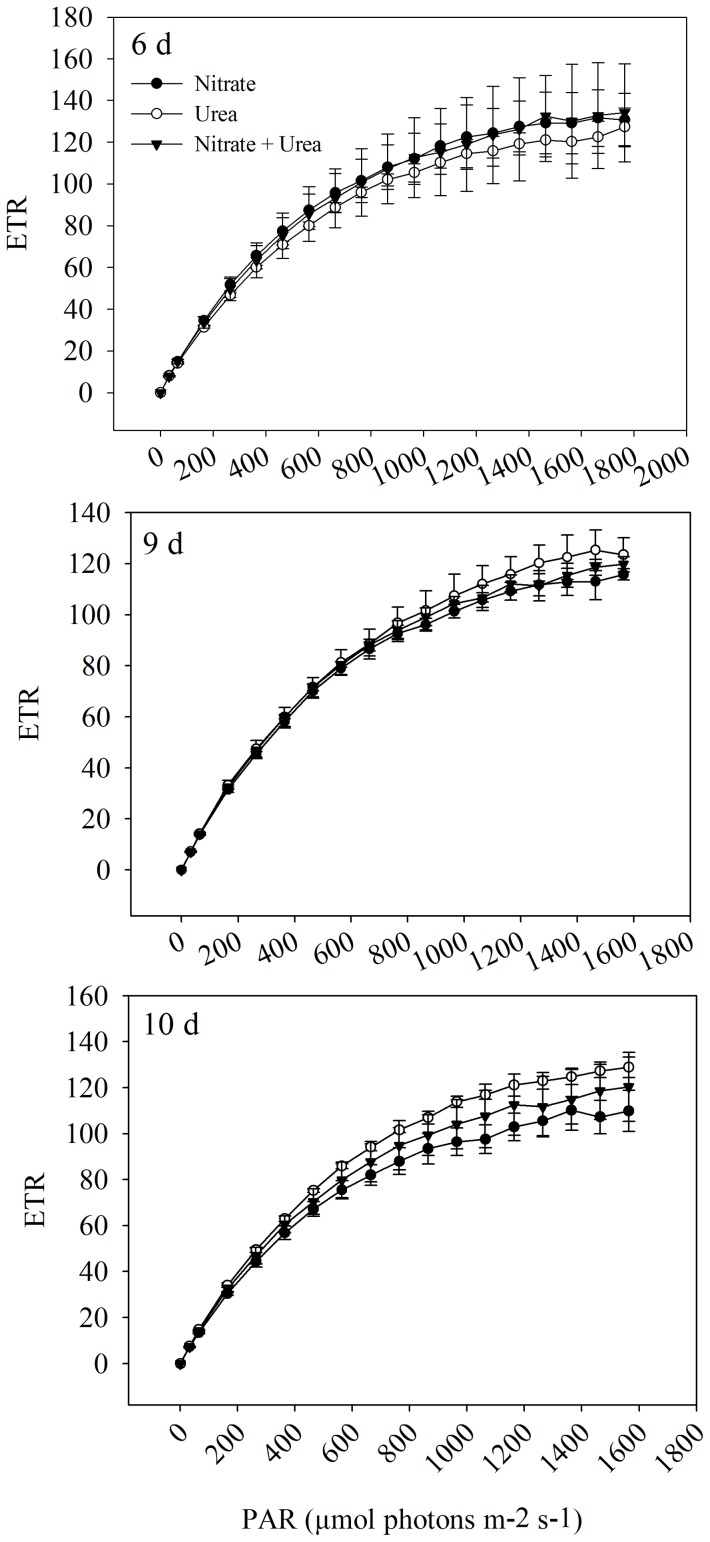
Relative electron transport rate (ETR) as a function of PAR. *A. anophagefferens* was grown on urea, nitrate, and a mixture of urea and nitrate, and samples were meaused at the 6-, 9- and 10-day. Error bars represent standard deviation of the mean for the three biological replicates.

**Figure 4:**
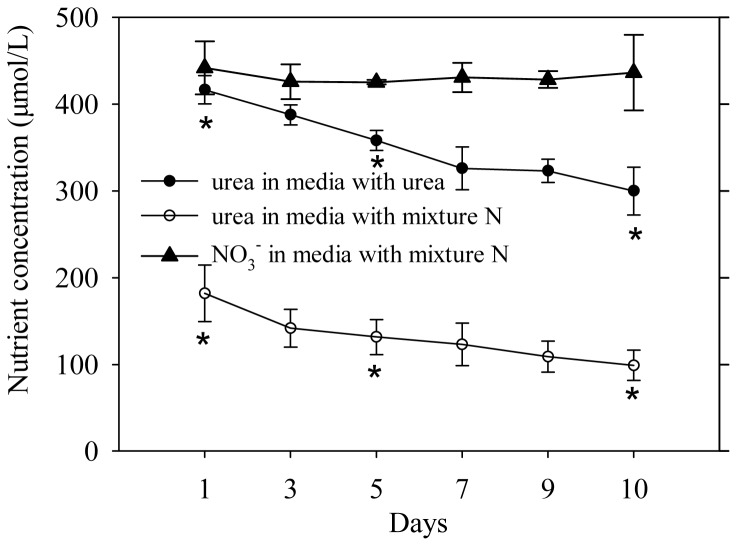
Concentrations of urea and nitrate as a function of culture time in media. Solid triangle represents nitrate concentration in medium with mixture N while solid and open circles represent urea concentrations in media with urea and mixture N, respectively. The difference between time points with asterisk was significant. Significance values were expressed as follows: * *P* <0.05, ** P <0.001. Error bars represent standard deviation of the mean for the three biological replicates.

For *A. anophagefferens* cells that were grown on different N sources, the cell density increased quickly during the first 5 days (exponential phase growth) (Fig. 1A). Maximum growth rates (calculated for days 1 to 5) were observed in cultures grown on urea (0.30 d^−1^), followed by the mixture of urea and nitrate (0.26 d^−1^), and nitrate (0.21 d^−1^) (Fig. 1C). However, there were no significant differences between the maximum growth rates. For cultures with nitrate, the cell density continued to increase after day 5, whereas the cell density did not increase in cultures with urea and mixture N. From day 7 to day 10, the cell density remained at the same level, and the density in all cultures tended to be consistent. The Fv/Fm (Maximum photochemical efficiency of PSII) of cultures from days 6, 9 and 10 was determined, and no significant difference was observed among the three N sources (Fig. 2A). Interestingly, the light-saturated electron transport rate (ETRmax) and electron transport efficiency (ETE) tended to increase gradually from day 6 to day 10 in cultures with urea, whereas these values appeared to drop from day 6 to day 10 in cultures with nitrate and mixture N (Fig. 3). On day 10, ETRmax and ETE of cultures with urea were higher than those values of cultures with the other N sources. In addition, it is noted that the profile of urea concentration as a function of day in cultures with mixture N was extremely similar to that in cultures with urea, whereas no significant decrease was observed in NO_3_^−^ concentration in cultures with mixture N (Fig. 4), which suggested that *A. anophagefferens* may primarily utilize N from urea instead of nitrate in the mixture N media.

For N-limited and recovery experiments, the cell density of N-limited cells started decreasing after day 3, whereas that of N-replete cells continued increasing (Fig. 1B). On day 5, the Fv/Fm of N-limited cells (0.52) was lower than that of N-replete cells (0.60) (Fig. 2B). The Fv/Fm was recovered at 24 hr after the addition of either urea or nitrate to N-limited cultures, and this recovery was independent of the nitrogen sources (Fig. 2B).

### RNA-seq analysis

Deconvolution and filtering of raw reads yielded a mean of 6,938,798 reads (range: 6,539,842 to 7,242,083 reads) per individual RNA-seq library (Table 1). Subsequent alignment of the clean reads to the *A. anophagefferens* reference genome yielded a mean of 5,852,408 reads (84.3%) for each sample that mapped to at least one location in the *A. anophagefferens* genome (Table 1). However, of these mapped reads, only 47.4 to 50.9% of the total reads were mapped to the reference transcript for each sample (Table 2). This result indicated that approximately 30% of the total reads mapped to non-coding regions in the genome.

**Table 1:** Summary of RNA-seq sequencing data (mapping to the reference genome).

Samples	Total reads	Total mapped reads	Perfect match	< = 2bp mismatch	Unique match	Multi-position match	Total unmapped reads
Mixture N	7,242,083	6,107,412(84.33%)	4,739,972(65.45%)	1,367,440(18.88%)	4,687,001(64.72%)	1,420,411(19.61%)	1,134,671(15.67%)
Nitrate	7,216,883	6,062,505(84.00%)	4,638,395(64.27%)	1,424,110(19.73%)	4,778,646(66.21%)	1,283,859(17.79%)	1,154,378(16.00%)
Urea	7,050,236	5,927,783(84.08%)	4,585,450(65.04%)	1,342,333(19.04%)	4,688,413(66.50%)	1,239,370(17.58%)	1,122,453(15.92%)
Nrep	6,539,842	5,487,229(83.90%)	3,987,158(60.97%)	1,500,071(22.94%)	4,646,256(71.05%)	840,973(12.86%)	1,052,613(16.10%)
Ndep	6,581,716	5,577,020(84.74%)	4,101,357(62.31%)	1,475,663(22.42%)	4,779,171(72.61%)	797,849(12.12%)	1,004,696(15.26%)
Urecov	6,871,619	5,812,055(84.58%)	4,290,560(62.44%)	1,521,495(22.14%)	4,838,377(70.41%)	973,678(14.17%)	1,059,564(15.42%)
Nrecov	7,069,212	5,992,854(84.77%)	4,377,598(61.92%)	1,615,256(22.85%)	4,948,338(70.00%)	1,044,516(14.78%)	1,076,358(15.23%)

Nrep, Ndep, Urecov, and Nrecov refer to the nitrogen-replete, limited, urea recovery, and nitrate recovery samples, respectively.

**Table 2:** Summary of RNA-seq sequencing data (mapping to the reference transcript).

Samples	Total reads	Total mapped reads	Perfect match	< = 2bp mismatch	Unique match	Multi-position match	Total unmapped reads
Mixture N	7,242,083	3,631,779(50.15%)	2,847,747(39.32%)	784,032(10.83%)	2,875,798(39.71%)	755,981(10.44%)	3,610,304(49.85%)
Nitrate	7,216,883	3,639,577(50.43%)	2,804,173(38.86%)	835,404(11.58%)	2,959,722(41.01%)	679,855(9.42%)	3,577,306(49.57%)
Urea	7,050,236	3,590,932(50.93%)	2,797,158(39.67%)	793,774(11.26%)	2,936,461(41.65%)	654,471(9.28%)	3,459,304(49.07%)
Nrep	6,539,842	3,126,200(47.80%)	2,313,610 (35.38%)	812,590 (12.43%)	2,708,821 (41.42%)	417,379 (6.38%)	3413642 (52.20%)
Ndep	6,581,716	3,118,690(47.38%)	2,355,066(35.78%)	763,624(11.60%)	2,740,361(41.64%)	378,329(5.75%)	3,463,026(52.62%)
Urecov	6,871,619	3,457,608(50.32%)	2,613,980(38.04%)	843,628(12.28%)	2,953,008(42.97%)	504,600(7.34%)	3,414,011(49.68%)
Nrecov	7,069,212	3,445,784(48.74%)	2,565,769(36.29%)	880,015(12.45%)	2,894,006(40.94%)	551,778(7.81%)	3,623,428(51.26%)

Nrep, Ndep, Urecov, and Nrecov refer to the nitrogen-replete, limited, urea recovery, and nitrate recovery samples, respectively.

**Figure 5:**
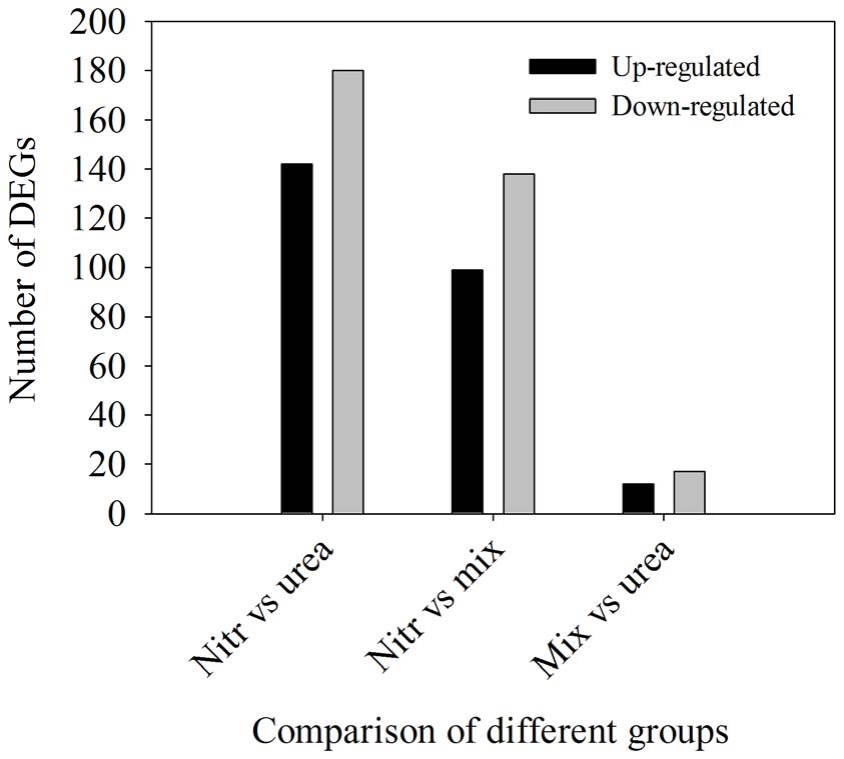
The number of differentially expressed genes among cells that were grown on the three N sources. Nitr, urea and mix represent cells grown on nitrate, urea and mixture N, respectively.

In *A. anophagefferens* grown on three different N sources, 9148 to 9526 genes were detected for each sample. The summary of the gene information is shown in Tables S1, S2 and S3, including unique read numbers that match each gene, its coverage, the expression level of each gene (represented by RPKM), and a putative function annotation. The data will be valuable for contributing to future genome annotation efforts and to the discovery of novel genes. A comparison of gene expression among the three N sources was performed. 322 differentially expressed genes were detected between nitrate-grown and urea-grown cells, 237 between nitrate-grown and mixture N-grown cells and 29 between urea-grown and mixture N-grown cells (Fig. 5, Table S4, S5 and S6). Fewer differentially expressed genes between urea-grown and mixture N-grown cells were identified, which further suggested the preferred utilization of cells for urea in media with mixture N.

For nitrogen-limited and recovery experiments, 707 genes were up-regulated significantly, and 766 were down-regulated significantly in N-depleted cells relative to N-replete cells. N-depleted cells exhibited a broad transcriptional response to nitrogen re-addition, with 681 genes up-regulated and 874 genes down-regulated in urea recovery cells, and 312 genes up-regulated and 688 genes down-regulated in nitrate recovery cells. The summary of those differentially expressed genes is listed in Table S7, S8 and S9.

### Nitrate versus urea

**Figure 6:**
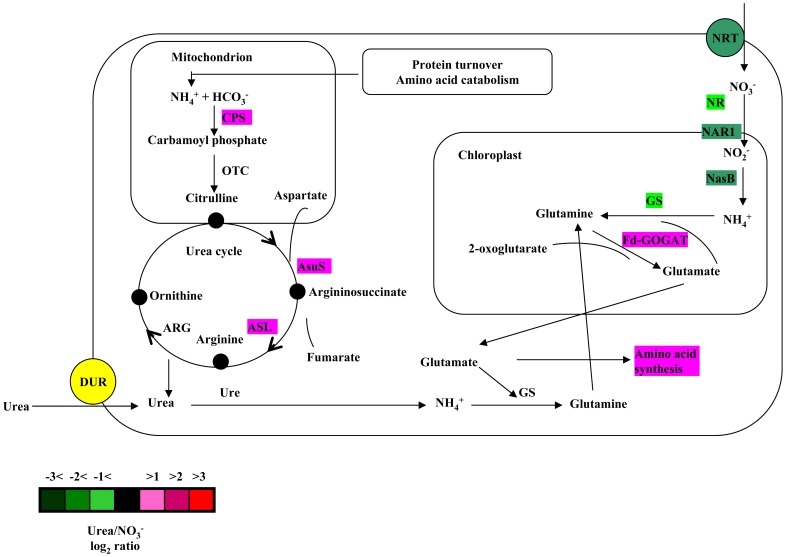
Proposed model showing the urea cycle, nitrate transport and assimilation, and the glutamine-glutamate cycle. Enzymes that are involved in these pathways are labeled with colors that indicate the fold change (log_2_) in their transcript levels in urea-grown cells relative to nitrate-grown cells (color code is provided in the figure). NRT, nitrate transporter; NR nitrate reductase; NAR1, nitrite transporter; NasB, NADPH nitrite reductase; GS, glutamine synthetase; Fd-GOGAT, ferredoxin-dependent glutamate synthase; CPS, carbamoyl phosphate synthase; OTC, ornithine transcarboxylase; AsuS, argininosuccinate synthase; ASL, argininosuccinate lyase; ARG, arginase; Ure, urease; DUR, urea transporter.

**Figure 7:**
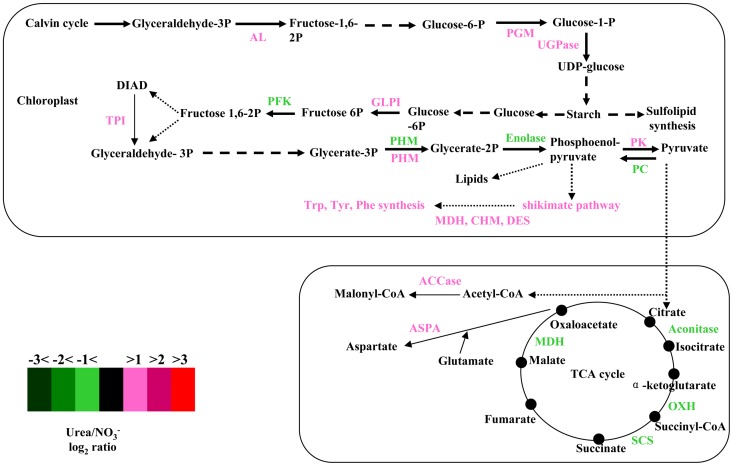
Pathways for starch synthesis, glycolysis, aromatic amino acid synthesis and the TCA cycle. Enzymes that are involved in these pathways are labeled with colors that indicate the fold change (log_2_) in their transcript levels in urea-grown cells relative to nitrate-grown cells (color code is provided in the figure). Dashed lines mean that no differentially expressed genes were detected in these pathways. AL, aldolase; PGM, phosphoglucomutase; UGPase, UDP-glucose-pyrophosphorylase; PFK, phosphofructokinase; TPI, triose-phosphate isomerase; PHM, phosphoglycerate mutase; PK, pyruvate kinase, PC, pyruvate carboxylase; OXH, oxoglutarate dehydrogenase; SCS, succinyl-CoA ligase; MDH, malate dehydrogenase; EPSPS, 3-phosphoshikimate 1-carboxyvinyltransferase; CHM, chorismate mutase; DES, dehydroquinate synthase; ASPA, aspartate aminotransferase; ACCase, Acetyl-CoA carboxylase; GLPI, glucose-6-phosphate isomerase.

Compared with cells that were grown on urea, 142 transcripts were upregulated 2-fold or greater in cells that were grown on nitrate, and 180 transcripts were downregulated 2-fold or greater in cells that were grown on nitrate (Table S4). Approximately 24% of differentially expressed genes could not be assigned a function because the reference genes represented hypothetical or predicted protein or showed no database homology. Transcripts encoding nitrate high affinity transporter, nitrite transporter NAR1, formate/nitrite transporter, and ammonium transporter increased when *A. anophagefferens* cells were grown on nitrate compared with urea. Transcripts encoding a putative nitrate reductase, NADPH nitrite reductase and glutamine synthetase increased in cells that were grown on nitrate compared with cells that were grown on urea (Fig. 6). Notably, 4 transcripts that were involved in OUC showed 2.0- to 2.6-fold upregulation in urea-grown cells relative to nitrate-grown cells (Table S4, Fig. 6). Thirty nine transcripts encoding proteins that were involved in protein synthesis showed 2.0- to 3.6-fold upregulation in urea-grown cells relative to nitrate-grown cells (Table S4). There were 11 transcripts encoding enzymes that were involved in amino acid synthesis that were upregulated in urea-grown cells relative to nitrate-grown cells. Levels of transcripts encoding proteins that were involved in photosynthesis and central carbon metabolism displayed difference in cells that were grown on urea and nitrate (Table S4, Fig. 7).

### Specific genes transcriptionally regulated by urea

**Figure 8:**
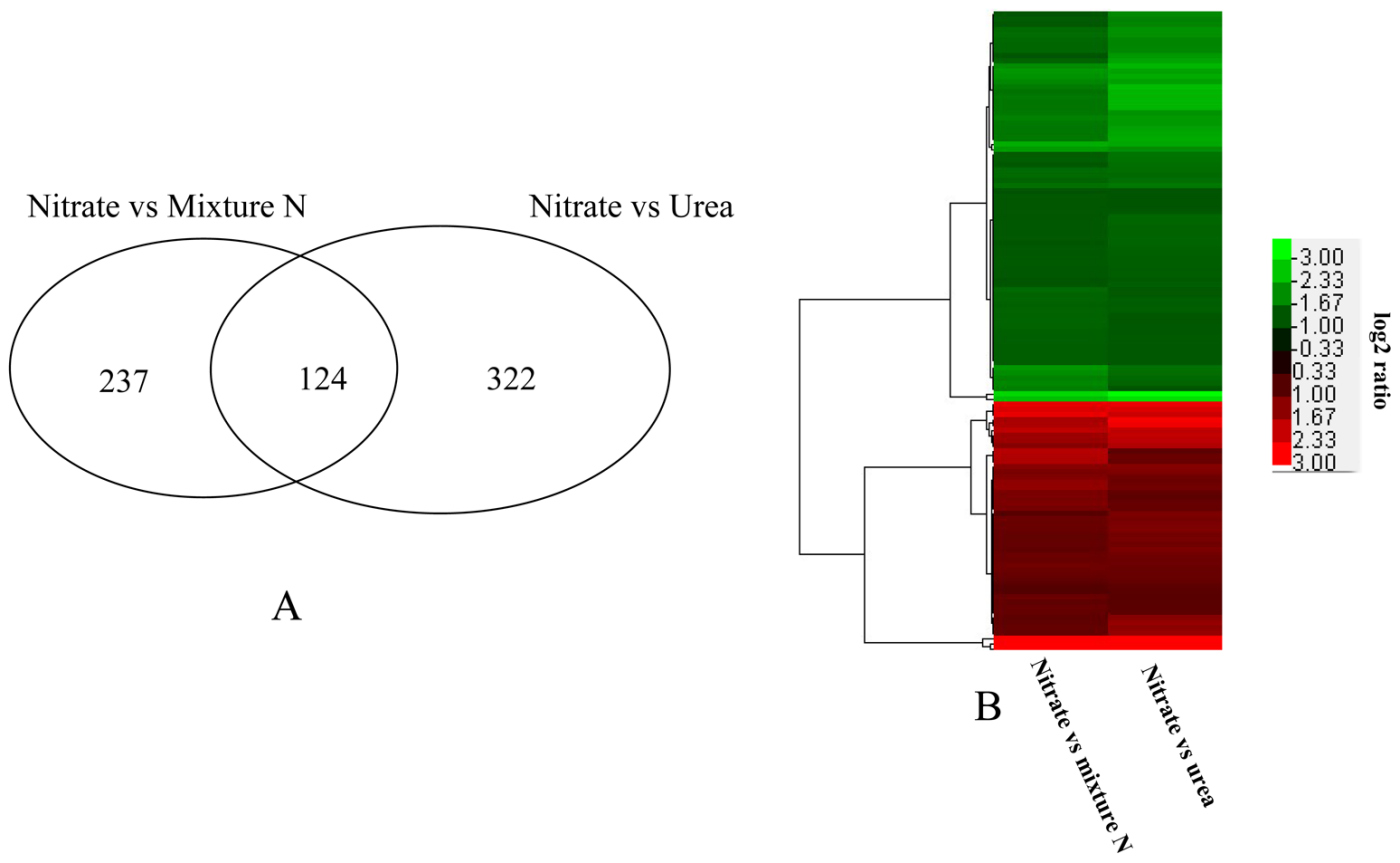
Specific genes transcriptionally regulated by urea. A) Number of common genes differentially regulated between the mixture N and urea groups compared with the reference nitrate group; B) the pattern of regulation of these common genes.

The comparison of results for the mixture N and urea groups with the nitrate reference allowed us to find genes for which transcription was specifically regulated by urea. We found 124 common genes differentially regulated between the mixture N and urea groups compared with the reference nitrate group (Figure 8A). These genes are listed in Table S10. Interestingly, the pattern of regulation of gene expression for urea and mixture N-grown cells appeared to be perfectly consistent (Figure 8B). Among these 124 genes, those involved in protein synthesis were well represented (Table 3). The next represented gene categories were nitrogen compound metabolism, protein modification and degradation, DNA and RNA binding, transport, signaling, photosynthesis, glycolysis/gluconeogenesis, and stress. Transcripts encoding argininosuccinate synthase, tryptophan synthase and spermine synthase increased in urea-and mixture-grown cells compared with nitrate-grown cells. Twenty two transcripts encoding proteins that are involved in protein synthesis were induced in urea-and mixture-grown cells.

**Table 3:** GO function class of the genes differentially regulated that are common between the urea and mixture N groups compared with the reference nitrate group.

Function class	Number of genes		
	Up-regulated^a^	Down-regulated^b^	Differentially expressed
Protein synthesis	22	0	22
Nitrogen compound metabolism	7	6	13
Transport	2	5	7
Glycolysis/gluconeogenesis	5	0	5
DNA and RNA (synthesis processing, transcription, regulation)	5	3	8
Photosynthesis	5	0	5
One-carbon compound metabolism	3	0	3
Protein (targeting, modification, degradation)	5	4	9
Signalling	2	4	6
Stress	2	3	5
Urea cycle	1	0	1
Sulfate assimilation	1	0	1
Citric acid cycle	0	1	1
Vanillin synthesis	0	1	1
Cell cycle regulation	0	2	2

Note: a and b represent number of genes that are differentially expressed in urea-and mixture-N cells compared with nitrate-grown cells.

### Nitrogen limitation and recovery

To evaluate the role of the *A. anophagefferens* OUC, RNA-seq was also used to investigate the pattern of gene expression of the OUC, TCA cycle and nitrogen assimilation pathway in response to nitrogen limitation and the addition of different nitrogen substrates to nitrogen-limited *A. anophagefferens* cultures. Under nitrogen-limited condition, two OUC-related transcripts and nine transcripts involved in nitrogen compound transport and assimilation showed upregulation, whereas three key transcripts for the TCA cycle were down-regulated (Table 4). In 24 h nitrogen-recovery experiments, all of the differentially expressed genes involved in these pathways that were significant were down-regulated (fold change >2.0 and *P*<0.05). No significant difference was observed in expression pattern of these transcripts between the addition of nitrate and urea.

**Table 4:** List of the genes differentially regulated among the nitrogen-replete (Nrep), limited (Ndep) and 24 h recovery (urea recovery, Urecov; nitrate recovery, Nrecov) libraries.

Metabolic pathway	Protein ID	Putative annotation	Ndep/Nrep		Urecov/Ndep		Nrecov/Ndep	
			log_2_ ratio	*P* value	log_2_ ratio	*P* value	log_2_ ratio	*P* value
Urea cycle	jgi|Auran1|20552	Carbamoyl-phosphate synthase	0.77	1.12E-164	-1.11	0	-1.82	0
	jgi|Auran1|33293	Ornithine carbamoyltransferase	0.70	3.33E-06	-1.00	1.26E-10	-0.84	3.35E-08
	jgi|Auran1|26092	Argininosuccinate synthase	1.06	8.24E-36	-0.26	3.55E-14	-0.87	8.00E-27
	jgi|Auran1|28200	Argininosuccinate lyase	0.61	8.78E-09	-0.17	0.08	-1.01	2.32E-20
	jgi|Auran1|72133	Urease	0.66	7.19E-91	-1.17	5.49E-245	-0.20	4.97E-11
	jgi|Auran1|30203	Urease accessory protein UreG	1.04	2.03E-08	-0.97	5.44E-08	-0.63	8.24E-4
Nitrogen assimilation pathway	jgi|Auran1|60332	Nitrate high affinity transporter	0.92	1.51E-101	-1.23	3.32E-171	-0.24	8.16E-11
	jgi|Auran1|53005	Nitrite transporter NAR1	-2.12	5.53E-112	-1.59	1.41E-19	-1.20	4.23E-13
	jgi|Auran1|17695	Formate/nitrite transporter	3.20	1.00E-29	-1.99	8.46E-19	-5.80	3.24E-45
	jgi|Auran1|15503	Formate/nitrite transporter	2.47	6.68E-61	-2.10	8.38E-53	-3.36	9.89E-87
	jgi|Auran1|55502	Xanthine uracil permease	1.52	0	-0.80	9.79E-116	-0.60	1.20E-69
	jgi|Auran1|53391	Nitrate reductase	1.33	4.40E-144	-1.18	1.05E-126	-1.13	2.77E-117
	jgi|Auran1|37238	NADPH nitrite reductase	1.41	3.41E-153	-4.22	0	-3.66	0
	jgi|Auran1|52709	NADH-dependent glutamate synthase	1.10	5.19E-57	-1.27	9.29E-75	-1.58	1.11E-101
	jgi|Auran1|38538	Ferredoxin-dependent glutamate synthase	0.62	2.52E-56	-0.82	7.50E-95	-1.39	1.86E-219
	jgi|Auran1|20700	Glutamine synthetase	1.25	4.90E-41	-1.20	1.40E-40	-0.57	3.68E-12
	jgi|Auran1|55217	Glutamine synthetase, type III	4.07	1.24E-08	-4.20	2.90E-09	-4.16	4.10E-09
	jgi|Auran1|60068	Formamidase	1.52	2.79E-05	-2.72	3.34E-10	-2.69	5.50E-10
	jgi|Auran1|21408	Cyanate lyase	3.64	5.79E-14	-3.09	6.70E-13	-3.06	1.21E-12
Citric acid cycle	jgi|Auran1|37323	Oxoglutarate dehydrogenase	-1.06	2.95E-57	-1.19	9.76E-36	-1.90	9.40E-69
	jgi|Auran1|28269	Succinyl-CoA ligase (SCS)	-0.74	5.08E-08	-0.66	1.15E-4	-1.37	1.35E-12
	jgi|Auran1|60302	Malate dehydrogenase (MDH)	-1.57	9.44E-77	-1.29	2.00E-21	-2.13	9.34E-42
	jgi|Auran1|59240	Isocitrate dehydrogenase (IDH)	-1.28	5.90E-226	0.74	7.43E-67	0.03	0.58

Log_2_ ratio >1 indicates that the gene is upregulated in nitrogen-limited and recovery conditions relative to the control. Log_2_ ratio <-1 indicates that the gene is downregulated in nitrogen-limited and recovery conditions relative to the control. Genes were considered differentally regulated at log_2_ ratio >1 and *P* <0.05.

## Discussion

In this study, RNA-Seq was used to profile the transcriptome of the Chinese strain of *A. anophagefferens* which was grown on urea, nitrate, and mixture N. Transcripts for the OUC, nitrogen assimilation, and TCA cycle in *A. anophagefferens* under nitrogen-limited and recovery conditions were also analyzed. Our goal is to gain a better understanding of the molecular mechanisms that underlie urea and nitrate metabolism in *A. anophagefferens* and to determine why *A. anophagefferens* prefers organic nitrogen to inorganic nitrogen on a molecular level.

### Nitrogen acquisition and assimilation

The levels of transcripts for several N transporters displayed significant differences between urea-grown and nitrate-grown cells (Table S4). Transcripts encoding nitrate high affinity transporter, nitrite transporter NAR1, formate/nitrite transporter, and ammonium transporter increased when *A. anophagefferens* cells were grown on nitrate compared with urea. The result suggested that these transporter genes are inducible by nitrate. In other phytoplankton, such as Chlorophyceae, Haptophyceae, and Bacillariophyceae, genes encoding high affinity nitrate transporters were highly induced when the cells were incubated with NO_3_^−^ or N starvation [22]. In addition, the relative expression of a putative nitrate transporter gene is higher in the American strain of *A. anophagefferens* that is grown on nitrate than in cells that are grown on other N sources [8]. Nitrate assimilation involves two membrane barriers, the plasma and the chloroplast membranes. Thus, once nitrate is reduced to nitrite in the cytosol, nitrite has to cross the chloroplast membranes for its subsequent reduction to ammonium [23]. In this study, transcripts for ID 53005 and 15503 had conservative domains of a formate/nitrite transporter and were homologous to the nitrite transporter NAR1 from *Ectocarpus siliculosus* and to a formate/nitrite transporter, respectively. The upregulation of these two transcripts suggested that the two genes might be involved in nitrite transport to chloroplasts. In *Chlamydomonas reinhardtii,* a Nar1 gene that encodes putative formate and nitrite transporters, has been found to play an important role in the regulation of nitrite transport to chloroplasts [24]. In *A. anophagefferens*, a higher fold change was observed in the abundance of the nitrite transporter NAR1, suggesting that the transporter (ID 53005) may be the main contributor to the regulation and transport of nitrite to the chloroplast. Interesting, transcript for the NAR1 was down-regulated under nitrogen limitation and recovery, whereas two transcripts for formate/nitrite transporters showed a strong upregulation under nitrogen limitation, and then sudden downregulation under short-term nitrogen recovery (Table 4). These results indicated that these nitrite transporter genes were regulated by nitrogen concentration. The rapid responses of these transporter genes to change of nitrate concentration imply that the Chinese strain of *A. anophagefferens* can utilize nitrate or nitrite effectively. In addition, it is expected that some of the genes such as the NAR1 (ID 53005) may be used for molecular biomarkers which are indicative of changes of the DIN during brown tides.

Nitrate also induced the upregulation of transcripts encoding a tryptophan/tyrosine permease and a xanthine/uracil/vitamin C permease (Table S4) other than ammonium transporter, which suggests that amino acids and nucleotides may be superior to nitrate as N sources for this strain. This trait is similar to that of the American strain of *A. anophagefferens* [8]. Notably, transcripts for xanthine uracil permease were upregulated in *A. anophagefferens* cells under N-limited condition (Table 4). The regulation of transcript for the xanthine uracil permease is consistent with a previous study on the American strain of *A. anophagefferens* [11]. These data suggest that the Chinese strain has the ability to utilize amino acids and nucleotides as N sources. This is consistent with other studies on the American strain of *A. anophagefferens* [5], [25].

Nitrate elicited an increase in the abundance of transcripts encoding proteins that are involved in NO3^−^ assimilation and in the synthesis of N metabolites (Table S4). Transcripts encoding a putative nitrate reductase, NADPH nitrite reductase and glutamine synthetase increased in cells that were grown on nitrate compared with cells that were grown on urea. Nitrate is not only an essential nutrient that activates the expression of genes for its assimilation pathway but also a signaling molecule that regulates cellular metabolism [26]. Our data describe how nitrate was transported and then reduced to ammonium in *A. anophagefferens* cells that were grown on nitrate (Fig. 6), and NADPH nitrite reductase may be the main contributor to the conversion of NO_2_^−^ into NH_4_^+^, instead of ferrdoxin nitrite reductase. Glutamine synthetase (GS) is one of the two enzymes that catalyze the glutamine-glutamate (GS-GOGAT) cycle. In diatoms, two GS isoenzymes have been characterized; GSII is localized to the chloroplast, whereas GSIII is cytosolic [27]. The gene encoding GSII is upregulated in diatom cells assimilating NO_3_^−^ but not in cells assimilating NH_4_^+^ taken up directly from the environment, where the gene encoding GSIII is constitutively expressed regardless of the presence or absence of nitrogen [28]. Based on these studies, we hypothesized that in *A. anophagefferens*, the GS that is encoded by the transcript (ID 20700) may be in chloroplasts, similar to the GSII of diatom, and can be induced by nitrate in media. Interestingly, no significant change was observed in the abundance of the GS transcript between cells that were grown on mixture N and that were grown on urea, which suggested that the presence of urea might inhibit the induction of the GS gene by the reduction of nitrate. Additionally, the results support the conclusion that *A. anophagefferens* primarily utilizes N from urea instead of nitrate in media with mixture N. Notably, the same three genes involved in nitrogen assimilation (ID 53391, 37238, 20700) induced by nitrate also were upregulated under nitrogen limitation and downregulated under nitrogen recovery (Table 4), suggesting that they were also regulated by nitrogen concentration.

Surprisingly, one transcript encoding a putative ferredoxin-dependent glutamate synthase (Fd-GOGAT) was shown to be upregulated in cells that were grown on urea relative to nitrate (Table S4). GOGAT catalyzes the transfer of the amide group from glutamine to 2-oxoglutarate to yield two molecules of glutamate (Fig. 6). One important fate of glutamate and glutamine is the synthesis of aspartate and asparagine. Evidence from higher plants demonstrates that the abundance of the Fd-GOGAT transcript is regulated by light but not by nitrogen sources [29]. However, to date, the regulation of the Fd-GOGAT gene in algae is largely unknown. This study is the first to find an increase in the Fd-GOGAT transcript in *A. anophagefferens* cells that were grown on urea relative to nitrate. In addition, no significant change was observed in the abundance of Fd-GOGAT transcript under nitrogen-limited condition (Table 4). The regulation of the gene deserves further study. Interestingly, a NADH-dependent glutamate synthase (NADH-GOGAT) was found to be upregulated under nitrogen limitation (Table 4). The data suggest that compared to Fd-GOGAT, NADH-GOGAT may play more important role in assimilation of ammonia released from intracellular nitrogen compounds in *A. anophagefferens* when N provision is limited.

In this study, two transcripts (ID 37987 and 60068) encoding putative formamidases were increased by 3- to 19-fold in cells that were grown on nitrate relative to urea (Table S4). An InterProScan Sequence Search showed that these transcripts contained a conservative domain of carbon-nitrogen hydrolase. Moreover, one transcript encoding a putative formamidase was shown to be upregulated in *A. anophagefferens* cells under N-limited conditions (Table 4). Increased activities of formamidase were detected in the American strain of *A. anophagefferens* under nitrogen depletion [10]. Our data implicate that this Chinese strain can break down small amides, which is consistent with studies for the American strain of *A. anophagefferens* [8]. Therefore, amides in seawaters may serve as N sources for field populations, especially those experiencing nitrogen depletion. It is noteworthy that one transcript encoding a putative cyanate lyase was increased by16-fold in *A. anophagefferens* cells under N-limited condition (Table 4). A putative cyanase gene has been identified in the American strain of *A. anophagefferens* [8]. The cyanate lyase can hydrolyze cyanate to ammonium and CO2. The upregulation of the transcript for cyanate lyase implicates that cyanate may also serve as a N source for field populations, especially those experiencing N starvation.

### The urea cycle

Interestingly, six genes encoding components of the OUC were found in our dataset including carbamoyl phosphate synthase (CPS), ornithine carbamoyltransferase (OTC), ornithine cyclodeaminase (OCD), argininosuccinate synthase (AsuS), argininosuccinate lyase (ASL), n-acetyl-gamma-glutamyl-phosphate reductase (AggPR). Among them, four transcripts increased in cells that were grown on urea relative to nitrate (Table S4, Fig. 6). Our results confirmed the presence of the OUC in *A. anophagefferens*. In metazoans, the OUC is involved in the catabolism of amino acids and in the generation of urea for export [30]. In diatoms, the OUC serves as a distribution and repackaging hub for inorganic carbon and nitrogen, suggesting that the diatom OUC is a key pathway for anaplerotic carbon fixation into nitrogenous compounds, which are essential for diatom growth [31]. In this study, the utilization of urea elicited an increase in the levels of transcripts encoding enzymes that are involved in the OUC, which contributes to the rapid repackaging and recycling of carbon and nitrogen from urea and protein catabolism. Compared with nitrate, the anaplerotic carbon fixation and rapid use for nitrogen through the OUC may be essential for the elevated growth of *A. anophagefferens* when given urea as a N source. It has been reported that for the American strain of *A. anophagefferens*, the culture with urea had a higher growth rate than the culture with other nitrogen sources tested [8]. However, in our study, growth of this Chinese strain on urea is not significantly faster than growth on nitrate. It is likely that the relative importance of different nutrient sources is variable, which may depend on availability and other conditions. Light intensity has been shown to be an important condition that regulates utilization of different nutrient sources in *A. anophagefferens* [7]. The influence of organic and inorganic nutrients can change over the course of a brown tide bloom, with varying effects depending on ambient nutrient levels [1]. In addition, the transcripts for the OUC were upregulated under nitrogen limitation and downregulated under nitrogen recovery, similar to the transcripts for nitrogen assimilation pathway (Table 4). The result indicated that the OUC-related genes in *A. anophagefferens* were transcriptionally regulated by nitrogen concentration, implying that they may be involved in anabolic metabolism of nitrogen and carbon in cells. In contrast, for *Arabidopsis thaliana* grown on different N sources, no transcriptional regulation by urea was observed for genes involved in the OUC cycle [32]. In total, it is postulated that for *A. anophagefferens,* the OUC cycle may play important roles in occurrence of blooms, especially when dissolved inorganic and organic nitrogen concentrations in seawater are changed.

Interestingly, we identified four transcripts encoding putative ureases that did not change significantly in abundance between cells that were grown on urea and nitrate (Tables S1 and S2). In the American strain of *A. anophagefferens*, urease activity varied positively with the growth rate, regardless of the N source [33]. Additionally, no significant change was observed in the abundance of the urease transcript in the American strain of *A. anophagefferens* cells under N deficiency (Table 4). Taken together, these results demonstrate that urease genes are constitutively expressed in both Chinese and American strain of *A. anophagefferens*, regardless of N concentration and N substrates. Consistent with its ecogenomic profile, ureases allow *A. anophagefferens* to meet its daily N demand from urea, whereas other phytoplankton do not [33].

**Figure 9:**
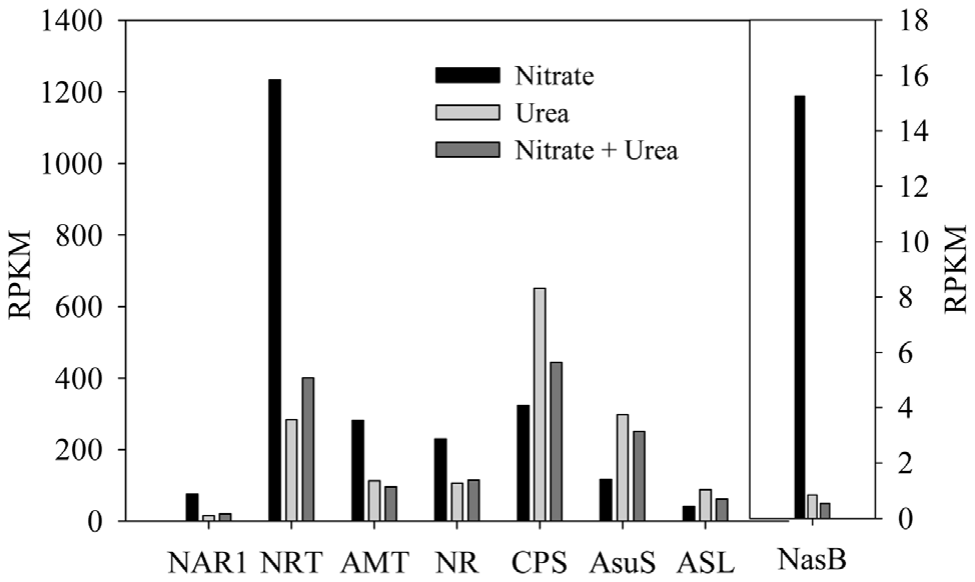
Absolute abundances of transcripts related to N assimilation pathway and the urea cycle. The transcript abundance that was obtained from RNA-seq data is indicated as RPKM (see Methods). The abundance of NasB transcript is shown on the right Y axis, while that of other transcripts is shown on the left Y axis. NAR1, nitrite transporter NAR1; NRT, nitrate high affinity transporter; AMT, ammonium transporter; NasB, NADPH nitrite reductase; NR, nitrate reductase; CPS, CPSase; AsuS, argininosuccinate synthase; ASL, argininosuccinate lyase.

Notably, in *A. anophagefferens* cells that are grown on mixture N, profiles of the absolute abundance of transcripts for nitrogen compound transport and metabolism, and the OUC were similar to those profiles of the same transcripts in cells that were grown on urea (Fig. 9). In particular, the absolute abundance of the transcript encoding NADPH nitrite reductase in nitrate-grown cells was approximately 18 times higher than that of the same transcript in urea and mixture N-grown cells (Fig. 9), suggesting that some substances in media with mixture N and urea markedly inhibit the expression of NADPH nitrite reductase gene. Moreover, as discussed above, a similar result was also observed in the GS transcript. Taken together, the results further support the observation that *A. anophagefferens* primarily feeds on urea instead of nitrate when urea and nitrate co-exist, which was obtained from nutrient changes in medium. This conclusion may be supported by the findings of Berg et al. (1997) that urea uptake constituted 58% to 64% of the total N uptake, whereas NO_3_^−^ uptake contributed between 5 and 8% [4]. Therefore, a possible mechanism is that NADPH nitrite reductase is inhibited by urea or its metabolic products in *A. anophagefferens* that is grown on mixture N.

### Amino acid and protein metabolism

Many transcripts encoding enzymes that are involved in amino acid synthesis increased in urea-grown cells relative to nitrate-grown cells (Table S4). For example, transcripts encoding two putative aspartate aminotransferases (ASMT) increased. This result was consistent with the observation in *Arabidopsis thaliana* that ASMT and Asp were increased in roots supplied with urea [32]. ASMT catalyzes the interconversion of aspartate and alpha-ketoglutarate to oxaloacetate and glutamate. Considering an increase in the GOGAT transcript, the elevated level of the ASMT transcript suggests that glutamate may positively regulate the expression of ASMT and direct the reaction to the synthesis of aspartate and alpha-ketoglutarate. In plants and microorganisms, aspartate is the precursor of several amino acids, including methionine, threonine, isoleucine, and lysine. In this study, three transcripts encoding enzymes that are involved in methionine metabolism and three transcripts encoding enzymes that are involved in S-adenosylmethionine (AdoMet) biosynthesis/recycling were shown to be increased in urea-grown cells. The methionine synthase transcript increased 3-fold in urea-grown cells compared with nitrate-grown cells. Methionine synthase not only catalyzes the last reaction in *de novo* methionine synthesis but also serves to regenerate the methyl group of AdoMet after methylation reactions [34]. In addition, two transcripts of methionine S-adenosyl transferase that catalyze the conversion of methionine to S-methyl-methionine also showed 3.9- to 4.7-fold upregulation in urea-grown cells (Table S4). Obviously, these results demonstrated that the synthesis of methionine and the activity of transmethylation were accelerated in urea-grown cells relative to nitrate-grown cells. Furthermore, one transcript encoding a putative ATP-sulfurylase, which is involved in sulfate assimilation, increased by 3.6-fold in urea-grown cells, which presumably indirectly supported the elevated synthesis of methionine and AdoMet. In plants, methionine occupies a central position in cellular metabolism, in which the processes of proteins, methyl-group transfers through AdoMet, and polyamines are interlocked [34]. We also identified two transcripts encoding spermine synthase and spermidine synthase that increased by 3.4- and 4.7-fold in urea-grown cells relative to nitrate-grown cells, respectively (Table S4). Spermine and spermidine are formed from AdoMet and the major polyamines in plants. Spermine and spermidine are involved in various processes, such as cell proliferation, growth, morphogenesis, differentiation, and programmed cell death [35]. Based on these results, it is likely that higher levels of AdoMet, spermine and spermidine promote the rapid growth of *A. anophagefferens* cells that are grown on urea.

Notably, three transcripts encoding enzymes that are involved in the synthesis of tryptophan, tyrosine, and phenylalanine through the shikimate pathway (Table S4, Fig. 7) and one transcript encoding tryptophan synthase increased in urea-grown cells relative to nitrate-grown cells (Table S4). In plants, these aromatic amino acids are not only essential components of protein synthesis but also serve as precursors for a wide range of secondary metabolites that are important for plant growth [36], [37]. It has been shown that the exposure of plants to various stresses generally induces the expression of genes that encode the shikimate pathway [37]. Therefore, in this study, the upregulation of transcripts that encode the shikimate pathway in urea-grown cells suggests that the utilization of organic N may boost the defensive ability of *A. anophagefferens* to abiotic and biotic stresses, such as predation of microbes and/or protistan grazers, and contribute to its rapid proliferation and blooms.

Surprisingly, many transcripts encoding ribosomal proteins and translation factors increased in urea-grown cells relative to nitrate-grown cells (Table S4). Meanwhile, genes involved in protein synthesis were the most abundant in the specific genes transcriptionally regulated by urea (Table 3). The results suggest that the sufficient provision of N in urea-grown cells elevated the capability of protein biosynthesis in these cells, which is consistent with an increase in amino acid biosynthesis and in transmethylation. The eukaryotic ribosome is not only responsible for protein synthesis but also plays a major role in controlling cell growth, division, and development [38]. Additionally, a positive correlation was observed between the level of ribosomal protein transcript accumulation and cell division in suspension culture cells [39]. In this study, although it is not clear which signal regulates the expression of ribosomal protein genes, the elevated level of 27 ribosomal protein transcripts may assist in accelerating the division and growth of *A. anophagefferens* cells.

Together, the expression difference of the genes for ribosomal proteins and synthesis of amino acids between urea-grown and nitrate-grown cells may be extended to the field of brown tides. It is hypothesized that the elevated expression levels of these genes may provide *A. anophagefferens* with a greater capacity to exploit organic nitrogenous compounds compared with other phytoplankton when inorganic nitrogen levels are low but organic nitrogen levels are elevated.

### Photosynthesis and carbon metabolism

In total, 57 transcripts that encode a putative plastid light harvesting protein were identified in *A. anophagefferens* (Table S1–S3). The genome of *A. anophagefferens* contains 62 genes that encode light-harvesting complex proteins (LHC), which is 1.5–3 times more than other eukaryotic phytoplankton that have been sequenced thus far [9]. LHC proteins bind antenna chlorophyll and carotenoid pigments, which increase the light-capturing capacity of photosynthetic reaction centers. In *Emiliania huxleyi*, LHC genes have been shown to increase under low light [40]. Our data demonstrated that 92% of these genes are useful and can be expressed at 100 µmol photon m^−2^ s^−1^ light, which confers a competitive advantage in absorbing light under low-irradiance conditions. In addition, of these 57 transcripts that encode LHC proteins, six transcripts were found to be upregulated in urea-grown cells relative to nitrate-grown cells and one displayed downregulation (Table S4), suggesting that different N sources may regulate different LHC genes. In combination with the profiles of Fv/Fm for *A. anophagefferens* that was grown on the three N sources (Fig. 2), these data demonstrated that changes in the abundance of a few LHC transcripts may not affect the ability of photosystems to capture light, suggesting that photosynthetic efficiency is independent of N sources when N provision is sufficient. This observation is consistent with the finding of Pustizzi et al. [7] that most of photosynthetic parameters were affected more by light intensity than by nitrogen source.

Interestingly, transcripts encoding two putative FNR (ID 23206 and 31888) increased in urea-grown cells, whereas transcript for another putative FNR (ID 52453) was markedly upregulated in nitrate-grown cells (Table S4). In the chloroplast, FNR catalyzes the interconversion between reduced ferredoxin and oxidized ferredoxin. When cells grow on nitrate, reduced ferredoxin is required for nitrite reductase (NiR) [41]. Thus, it is deduced that the gene encoding FNR (ID 52453) is induced by nitrate and works in reverse to provide reduced ferredoxin with nitrate reduction. Based on rapid light curves on day 10, ETRmax and ETE of cells grown on urea were higher than those values of cells that were grown on other N sources. The genes encoding FNRs (ID 23206 and 31888) may be the main contributors to the transfer of electron from reduced ferredoxin to NADPH during photosynthesis. It is deduced that *A. anophagefferens* cells that were grown on urea were more efficient at maintaining the expression level of genes encoding FNRs (ID 23206 and 31888) in the late phase of growth, whereas cells that were grown on nitrate were not. This observation may be due to the competition for electrons between NADP^+^ photoreduction and ferredoxin-dependent N assimilation in cells that were grown on nitrate.

Transcripts encoding key proteins of starch synthesis increased in urea-grown cells, whereas transcripts encoding key proteins of the TCA cycle increased in nitrate-grown cells (Fig. 7). For glycolysis, one transcript encoding a putative phosphofructokinase, which is involved in a key regulatory step in the glycolytic pathway, increased by 2.2-fold in nitrate-grown cells, whereas two transcripts encoding putative pyruvate kinase increased by 3.0- to 3.2-fold in urea-grown cells. These enzymes are specific to glycolysis. In addition, other transcripts encoding glucose-6-phosphate isomerase, enolase, triose-phosphate isomerase and pyruvate carboxylase, which are involved in glycolysis and gluconeogenesis, were upregulated in urea-grown or nitrate-grown cells. These results may suggest that glycolytic activity changes little in cells that are grown on different nitrogen sources. However, relative to urea-grown cells, the activity of the TCA cycle was elevated in nitrate-grown cells. The TCA cycle is not only a source of energy and reducing equivalents but also provides carbon skeletons for nitrogen assimilation and for the biosynthesis of compounds. Along with an increase in starch synthesis in urea-grown cells, this result suggests that more carbon from the Calvin cycle may be channeled into the TCA cycle in nitrate-grown cells relative to urea-grown cells. In contrast, more carbon from the Calvin cycle may accumulate as starch in urea-grown cells relative to nitrate-grown cells. This difference may be due to the elevated activity of the OUC in urea-grown cells. In diatoms, correlation analyses of metabolites of the TCA cycle and OUC indicated that the OUC derivatives urea and proline are tightly coupled to TCA cycle intermediates, which suggests that there are important connections between the OUC, the glutamine/glutamate cycle and the TCA cycle in diatoms [31]. In this study, the differential expression of genes for the OUC, the TCA cycle, and the glutamine/glutamate cycle suggested that, similar to animal cells [42], the OUC may be linked to the TCA cycle through the aspartate-argininosuccinate shunt (Fig. 6) in *A. anophagefferens* cells. Different patterns of transcripts for the OUC and TCA cycle in *A. anophagefferens* cells under N-limited and recovery conditions may also support the conclusion. Perhaps, the anaplerotic carbon-fixation for urea-C through the OUC attenuates the activity of the TCA cycle and leads to the accumulation of starch. This turnover and reallocation of intracellular carbon and nitrogen into key cellular components, such as protein, AdoMet, spermine and aromatic amino acids, may increase competitiveness of *A. anophagefferens* relative to other phytoplankton when concentrations of dissolved organic nitrogen are elevated in the anthropogenically coastal waters.

### Signal transduction

Transcripts encoding many putative signal proteins, such as (p)ppGpp synthetase I, G protein, protein kinases, RNA-binding region RNP-1, Nog1 nucleolar GTPase, proteins containing the WD 40 domain, phospholipase D, and RAB family GTPase were shown to be upregulated in urea-grown or nitrate-grown cells (Table S4). These genes may synergistically mediate elaborate cell signaling and density sensing in blooms, which is important for detecting an ambient environment. Based on these data, it is difficult to distinguish the significance of the differential expression of genes that are involved in signal transduction, which are regulated by urea or nitrate. However, as has been reported, the *A. anophagefferens* genome encodes many more proteins that are involved in cell signaling transduction than other phytoplankton [9]. Our study demonstrated the expression of these genes and suggested that these genes may play important roles in the formation of blooms.

Notably, five transcripts encoding enzymes that regulate the biosynthesis of sterols increased in urea-grown cells relative to nitrate-grown cells (Table S4). In higher plants, sterols are precursors of steroid hormones. It is reported that, in *Arabidopsis*, sitosterol, stigmasterol, and some abnormal sterols upregulate the characteristic cell expansion and proliferation of genes [43]. Sterols themselves may act as signaling molecules in plants in a manner that is analogous to the action of cholesterol in mammalian systems [44]. In this study, an increase in transcript for the biosynthesis of sterols may provide cells with sufficient sterols and steroid hormones, which may promote cell expansion and proliferation. Interestingly, one transcript encoding a putative pescadillo-like protein was shown to increase 3.3-fold in urea-grown cells relative to nitrate-grown cells (Table S4). In yeast, pescadillo plays a crucial role in cell proliferation and in the cell cycle [45]. Therefore, in *A. anophagefferens*, the putative pescadillo-like protein may be a key regulatory protein that affects cell proliferation and cell cycle progression.

### Comparisons between the Chinese and American strains

This Chinese strain we isolated has been reported to have 99.7–100% similarity to *A. anophagefferens* Hargraves et Sieburth, the causative species of brown tides on the east coast of USA based on the 18S rDNA [2]. In addition, characteristic pigment, 19′-butanoyloxyfucoxanthin in the American strain has also been detected in the Chinese strain of *A. anophagefferens* [46]. For *A. anophagefferens* grown on urea, nitrate, and the mixture of urea and nitrate, some physiological features of the Chinese strain including the growth rate and Fv/Fm were similar to non-axenic cultures of the American strain [7]. However, the maximum growth rate of the Chinese strain was lower than that of the American strain [7].

Our RNA-seq data showed that 83.9–84.7% of the clean reads from the Chinese strain mapped to at least one location in genome of the American strain of *A. anophagefferens* for each sample. Many genes encoding proteins that were involved in nitrogen acquisition and assimilation in the Chinese strain exhibited similar responses with the American strain to nitrate, urea and nitrogen depletion. These data further indicate that the Chinese strain has genetically high similarity to the American strain of *A. anophagefferens.* In addition, we performed a systems-level analysis of the expression differences of genes regulated by urea or nitrate in the Chinese strain. Changes of some important metabolic pathways including the OUC, TCA cycle, and amino acid synthesis were highlighted. To date, these systemic analyses are not reported in the American strain.

We also noted that the reference transcript we used is not pure transcriptome of *A. anophagefferens*, but a dataset that includes complete gene models predicted from *A. anophagefferens* genome and from available EST and cDNA data. However, only 47.4 to 50.9% of the reads mapped to the gene models in the dataset for each sample. For these reads that did not map to any gene models in the reference transcript, but mapped to the genome sequence, one possible explanation is that they may be associated with exon-3′ UTR and exon-5′ UTR sequences, intergenic locations and intronic regions. As for the reads that did not map to any location in the genome of the American strain of *A. anophagefferens*, this may be due to the gene differences between the Chinese and American strains or errors in gene predictions.

## Conclusions

In this study, similar levels of transcripts for nitrate transport and assimilation were detected in mixture N-grown cells and urea-grown cells. Together with changes in nutrient concentrations in media, these results may suggest that *A. anophagefferens* primarily feeds on urea instead of nitrate when urea and nitrate co-exist. A possible mechanism is that NADPH nitrite reductase is inhibited by urea or its metabolic products in urea-grown cells. Transcripts for the OUC, and synthesis of glutamate and aspartate were upregulated by urea, whereas transcripts for the TCA cycle were negatively regulated by urea treatment, suggesting that in *A. anophagefferens* cells, the OUC may be linked to the TCA cycle through the aspartate-argininosuccinate shunt. This speculation was further supported by pattern of transcripts for the OUC and TCA cycles in response to N-limitation and recovery. Transcripts for the biosynthesis of sterols and pescadillo were markedly upregulated in urea-grown cells, presumably regulating the rapid proliferation of *A. anophagefferens* cells and blooms. This study is the first to determine potential roles of the OUC in the reallocation of intracellular carbon and nitrogen in *A. anophagefferens* cells. Our results may provide a partial explanation for blooms of *A. anophagefferens* in estuaries with elevated levels of organic matter.

## Acknowledgments

We thank BGI-tech (Shenzhen) for sequencing and Y. Zhang for bioinformatic analysis.

## Supporting Information

Table S1 The information of genes identified in *A. anophagefferens* grown on urea using RNA-seq technology.
(XLS)

Table S2 The information of genes identified in *A. anophagefferens* grown on nitrate using RNA-seq technology.
(XLS)

Table S3 The information of genes identified in *A. anophagefferens* grown on mixture N using RNA-seq technology.
(XLS)

Table S4 Information of differentially expressed genes in *A. anophagefferens* cells identified when nitrate-grown cells were compared to urea-grown cells.
(XLS)

Table S5 Information of differentially expressed genes in *A. anophagefferens* cells identified when nitrate-grown cells were compared to mixture N-grown cells.
(XLS)

Table S6 Information of differentially expressed genes in *A. anophagefferens* cells identified when mixture N-grown cells were compared to urea-grown cells.
(XLS)

Table S7 Information of differentially expressed genes in *A. anophagefferens* cells identified when N-depleted cells were compared to N-replete cells.
(XLS)

Table S8 Information of differentially expressed genes in *A. anophagefferens* cells identified when urea-recovery cells were compared to N-depleted cells.
(XLS)

Table S9 Information of differentially expressed genes in *A. anophagefferens* cells identified when nitrate-recovery cells were compared to N-depleted cells.
(XLS)

Table S10 Information of common differentially expressed genes in *A. anophagefferens* cells identified when nitrate-grown cells were compared to mixture N-grown and urea-grown cells, respectively.
(XLS)

## References

[1] Gobler CJ, Lonsdale DJ, Boyer GL (2005) A review of the causes, effects, and potential management of harmful brown tide blooms caused by *Aureococcus anophagefferens* (Hargraves et sieburth). Estuaries 28: 726–749.

[2] Zhang QC, Qiu LM, Yu RC, Kong FZ, Wang YF, et al (2012) Emergence of brown tides caused by *Aureococcus anophagefferens* Hargraves et Sieburth in China. Harmful Algae 19: 117–124.

[3] Anderson DM, Burkholder JM, Cochlan WP, Glibert PM, Gobler CJ, et al (2008) Harmful algal blooms and eutrophication: Examining linkages from selected coastal regions of the United States. Harmful Algae 8: 39–53. 19956363 10.1016/j.hal.2008.08.017 PMC2677713

[4] Berg GM, Glibert PM, Lomas MW, Burford MA (1997) Organic nitrogen uptake and growth by the chrysophyte *Aureococcus anophagefferens* during a brown tide event. Mar Biol 129: 377–387.

[5] Mulholland MR, Gobler CJ, Lee C (2002) Peptide hydrolysis, amino acid oxidation, and nitrogen uptake in communities seasonally dominated by *Aureococcus anophagefferens* . Limnol Oceanogr 47: 1094–1108.

[6] Lomas MW, Glibert PM, Clougherty DA, Huber DR, Jones J, et al (2001) Elevated organic nutrient ratios associated with brown tide algal blooms of *Aureococcus anophagefferens* (Pelagophyceae). J Plankton Res 23: 1339–1344.

[7] Pustizzi F, MacIntyre H, Warner ME, Hutchins DA (2004) Interaction of nitrogen source and light intensity on the growth and photosynthesis of the brown tide alga *Aureococcus anophagefferens* . Harmful Algae 3: 343–360.

[8] Berg GM, Shrager J, Glöckner G, Arrigo KR, Grossman AR (2008) understanding nitrogen limitation in *Aureococcus anophagefferens* (Pelagophyceae) through cDNA and qRT-PCR analysis. J Phycol 44: 1235–1249. 27041720 10.1111/j.1529-8817.2008.00571.x

[9] Gobler CJ, Berry DL, Dyhrman ST, Wilhelm SW, Salamov A, et al (2011) Niche of harmful alga *Aureococcus anophagefferens* revealed through ecogenomics. P Nat Acad Sci 108: 4352–4357. 10.1073/pnas.1016106108 PMC3060233 21368207

[10] Gobler CJ, Berry DL, Dyhrman ST, Wilhelm SW, Salamov A, et al (2011) Niche of harmful alga *Aureococcus anophagefferens* revealed through ecogenomics. P Nat Acad Sci USA 108: 4352–4357. 10.1073/pnas.1016106108 PMC3060233 21368207

[11] Wurch LL, Haley ST, Orchard ED, Gobler CJ, Dyhrman ST (2011) Nutrient-regulated transcriptional responses in the brown tide-forming alga *Aureococcus anophagefferens* . Environ Microbiol 13: 468–481. 20880332 10.1111/j.1462-2920.2010.02351.x PMC3282463

[12] Wang Z, Gerstein M, Snyder M (2009) RNA-Seq: a revolutionary tool for transcriptomics. Nat Rev Genet 10: 57–63. 19015660 10.1038/nrg2484 PMC2949280

[13] Gonzalez-Ballester D, Casero D, Cokus S, Pellegrini M, Merchant SS, et al (2010) RNA-Seq Analysis of Sulfur-Deprived Chlamydomonas Cells Reveals Aspects of Acclimation Critical for Cell Survival. Plant Cell 22: 2058–2084. 20587772 10.1105/tpc.109.071167 PMC2910963

[14] Goldman JC, McCarthy JJ (1978) Steady state growth and ammonium uptake of a fast-growing marine diatom. Limnol Oceanogr 23: 695–703.

[15] Webb W, Newton M, Starr D (1974) Carbon dioxide exchange of Alnus rubra. Oecologia 17: 281–291. 28308943 10.1007/BF00345747

[16] Rahmatullah M, Boyde TRC (1980) Improvements in the determination of urea using diacetyl monoxime; methods with and without deproteinisation. Clinica Chimica Acta 107: 3–9. 10.1016/0009-8981(80)90407-6 7428175

[17] Anderson L (1979) Simultaneous spectrophotometric determination of nitrite and nitrate by flow injection analysis. Anal Chim Acta 110: 123–128.

[18] Li R, Yu C, Li Y, Lam TW, Yiu SM, et al (2009) SOAP2: an improved ultrafast tool for short read alignment. Bioinformatics 25: 1966–1967. 19497933 10.1093/bioinformatics/btp336

[19] Morrissy AS, Morin RD, Delaney A, Zeng T, McDonald H, et al (2009) Next-generation tag sequencing for cancer gene expression profiling. Genome Res 19: 1825–1835. 19541910 10.1101/gr.094482.109 PMC2765282

[20] Chen S, Yang P, Jiang F, Wei Y, Ma Z, et al (2010) *De Novo* Analysis of Transcriptome Dynamics in the Migratory Locust during the Development of Phase Traits. PloS one 5: e15633. 21209894 10.1371/journal.pone.0015633 PMC3012706

[21] Audic S, Claverie JM (1997) The significance of digital gene expression profiles. Genome Res 7: 986–995. 9331369 10.1101/gr.7.10.986

[22] Song B, Ward BB (2007) Molecular cloning and characterization of high-affinity nitrate transporters in marine phytoplankton. J Phycol 43: 542–552.

[23] Crawford NM (1995) Nitrate: nutrient and signal for plant growth. Plant Cell 7: 859–868. 7640524 10.1105/tpc.7.7.859 PMC160877

[24] Rexach J, Fernandez E, Galvan A (2000) The *Chlamydomonas reinhardtii* Nar1 Gene Encodes a Chloroplast Membrane Protein Involved in Nitrite Transport. Plant Cell 12: 1441–1453. 10948261 10.1105/tpc.12.8.1441 PMC149114

[25] Berg GM, Repeta DJ, Laroche J (2002) Dissolved Organic Nitrogen Hydrolysis Rates in Axenic Cultures of *Aureococcus anophagefferens* (Pelagophyceae): Comparison with Heterotrophic Bacteria. Appl environ microb 68: 401–404. 10.1128/AEM.68.1.401-404.2002 PMC126575 11772651

[26] Fernandez E, Galvan A (2008) Nitrate Assimilation in Chlamydomonas. Eukaryotic Cell 7: 555–559. 18310352 10.1128/EC.00431-07 PMC2292633

[27] Armbrust EV, Berges JA, Bowler C, Green BR, Martinez D, et al (2004) The Genome of the Diatom *Thalassiosira Pseudonana*: Ecology, Evolution, and Metabolism. Science 306: 79–86. 15459382 10.1126/science.1101156

[28] Takabayashi M, Wilkerson FP, Robertson D (2005) response of glutamine synthetase gene transcription and enzyme to external nitrogen sources in the diatom *Skeletonema Costatum* (Bacillariophyceae). J Phycol 41: 84–94.

[29] Coschigano KT, Melo-Oliveira R, Lim J, Coruzzi GM (1998) Arabidopsis gls Mutants and Distinct Fd-GOGAT Genes: Implications for Photorespiration and Primary Nitrogen Assimilation. Plant Cell 10: 741–752. 9596633 10.1105/tpc.10.5.741 PMC144371

[30] Lee B, Yu H, Jahoor F, O'Brien W, Beaudet AL, et al (2000) In vivo urea cycle flux distinguishes and correlates with phenotypic severity in disorders of the urea cycle. P Nat Acad of Sci 97: 8021–8026. 10.1073/pnas.140082197 PMC16663 10869432

[31] Allen AE, Dupont CL, Obornik M, Horak A, Nunes-Nesi A, et al (2011) Evolution and metabolic significance of the urea cycle in photosynthetic diatoms. Nature 473: 203–207. 21562560 10.1038/nature10074

[32] Merigout P, Lelandais M, Bitton F, Renou J-P, Briand X, et al (2008) Physiological and transcriptomic aspects of urea uptake and assimilation in Arabidopsis Plants. Plant Physiology 147: 1225–1238. 18508958 10.1104/pp.108.119339 PMC2442537

[33] Fan C, Glibert PM, Alexander J, Lomas MW (2003) Characterization of urease activity in three marine phytoplankton species, *Aureococcus anophagefferens*, Prorocentrum minimum, and Thalassiosira weissflogii. Mar Biol 142: 949–958.

[34] Ravanel S, Gakiere B, Job D, Douce R (1998) The specific features of methionine biosynthesis and metabolism in plants. P Nat Acad Sci USA 95: 7805–7812. 10.1073/pnas.95.13.7805 PMC22764 9636232

[35] Kumar A, Taylor M, Altabella T, Tiburcio AF (1997) Recent advances in polyamine research. Trends Plant Sci 2: 124–130.

[36] Dixon RA (2001) Natural products and plant disease resistance. Nature 411: 843–847. 11459067 10.1038/35081178

[37] Tzin V, Galili G (2010) new insights into the shikimate and aromatic amino acids biosynthesis pathways in plants. Molecular Plant 3: 956–972. 20817774 10.1093/mp/ssq048

[38] Ito T, Kim G-T, Shinozaki K (2000) Disruption of an Arabidopsis cytoplasmic ribosomal protein S13-homologous gene by transposon-mediated mutagenesis causes aberrant growth and development. Plant J 22: 257–264. 10849343 10.1046/j.1365-313x.2000.00728.x

[39] Gao J, Kim S-R, Chung Y-Y, Lee J, An G (1994) Developmental and environmental regulation of two ribosomal protein genes in tobacco. Plant Mol Biol 25: 761–770. 8075394 10.1007/BF00028872

[40] Lefebvre SC, Harris G, Webster R, Leonardos N, Geider RJ, et al Characterization and expression analysis of the Lhcf gene family in *Emiliania Huxleyi* (Haptophyta) reveals differential responses to light and CO_2_ . J Phycol 46: 123–134.

[41] Brunswick P, Cresswell CF (1988) Nitrite uptake into intact pea chloroplasts: I. kinetics and relationship with nitrite assimilation. Plant Physiol 86: 378–383. 16665916 10.1104/pp.86.2.378 PMC1054492

[42] Morris SM (2002) Regulation of enzymes of the urea cycle and arginine metabolism. Annu Rev Nutr 22: 87–105. 12055339 10.1146/annurev.nutr.22.110801.140547

[43] He J-X, Fujioka S, Li T-C, Kang SG, Seto H, et al (2003) Sterols regulate development and gene expression in Arabidopsis. Plant Physiol 131: 1258–1269. 12644676 10.1104/pp.014605 PMC166886

[44] Carland F, Fujioka S, Nelson T (2010) The sterol methyltransferases SMT1, SMT2, and SMT3 influence Arabidopsis development through nonbrassinosteroid products. Plant Physiol 153: 741–756. 20421456 10.1104/pp.109.152587 PMC2879779

[45] Kinoshita Y, Jarell AD, Flaman JM, Foltz G, Schuster J, et al (2001) Pescadillo, a novel cell cycle regulatory protein abnormally expressed in malignant cells. J Biol Chem 276: 6656–6665. 11071894 10.1074/jbc.M008536200

[46] Kong F, Yu R, Zhang Q, Yan T, Zhou M (2012) Pigment characterization for the 2011 bloom in Qinhuangdao implicated “brown tide” events in China. Chin J Oceanol Limn 30: 361–370.

